# Laser-Directed Energy Deposition of Fe-Mn-Si-Based Shape Memory Alloy: Microstructure, Mechanical Properties, and Shape Memory Properties

**DOI:** 10.3390/ma17010131

**Published:** 2023-12-27

**Authors:** Bing Liu, Cong Yao, Jingtao Kang, Ruidi Li, Pengda Niu

**Affiliations:** 1 Wuhan Second Ship Design and Research Institute, Wuhan 430064, China; 2 State Key Laboratory of Powder Metallurgy, Central South University, Changsha 410083, China

**Keywords:** laser-directed energy deposition (LDED), Fe-Mn-Si-based shape memory alloy, stress-induced martensite, mechanical properties, shape memory properties

## Abstract

Fe-Mn-Si shape memory alloys (SMAs) have gained significant attention due to their unique characteristics. However, there remains a gap in the literature regarding the fabrication of these alloys using laser-directed energy deposition (LDED). This study fills this void, investigating the properties of Fe-Mn-Si SMAs produced by LDED. The shape memory performance of as-deposited Fe-Mn-Si SMAs was studied using a tensile method. Alloys underwent different degrees of deformation to assess their shape memory effect. Microstructural evaluations were conducted post-deformation to observe the internal structures of the alloys. The tensile tests revealed that shape recovery rates for deformation levels of 3%, 7%, 11%, and 15% were 68.1%, 44.2%, 31.7%, and 17.6%, respectively. Notably, the maximum recoverable deformation of the LDED-formed Fe-Mn-Si-based shape memory alloy reached 3.49%, surpassing the traditional deformation processing SMAs (<3%). The presence of a significant number of stacking faults was linked to the enhanced shape memory performance. The LDED technique demonstrates promising potential for the fabrication of Fe-Mn-Si SMAs, producing alloys with enhanced shape memory performance compared to traditionally processed SMAs. The study’s findings offer new insights and broaden the applicability of LDED in the field of SMAs.

## 1. Introduction

Shape memory alloys (SMAs) are distinguished by their ability to regain their original shape after undergoing significant deformation when subjected to an external stimulus, such as heat. This unique property has propelled extensive research into various SMA materials for diverse applications across sectors including aerospace, automotive, and biomedical industries [1,2,3]. Amongst the array of SMAs, Fe-Mn-Si-based alloys have attracted considerable interest due to their cost-effectiveness, excellent mechanical properties, and particularly, their potential for significant ductility and high damping capacity [4,5,6,7,8].

The traditional processing of Fe-Mn-Si-based SMAs typically involves conventional metallurgical methods such as casting, rolling, and heat treatment [9]. Research into these conventional processes has focused extensively on understanding the thermomechanical behaviors and the effects of alloying elements on the martensitic transformation, which is fundamental to the shape memory effect. Studies have shown that the addition of elements like Cr and Ni can significantly influence phase transformation temperatures, improving mechanical properties and corrosion resistance [8,10]. Especially, adding V and C elements can improve pseudo-elasticity via decreasing the grain size and precipitation of VCs [11,12]. However, these conventional methods often involve cumbersome processes and could lead to inhomogeneous microstructures, which limits the improvement of the shape memory effect.

In recent years, additive manufacturing (AM) has emerged as a groundbreaking alternative in the fabrication of SMAs, providing unprecedented capabilities for producing components with intricate geometries and customized mechanical properties. Among the many additive manufacturing technologies, LPBF has proven capable of forming precise and complex parts in materials such as titanium alloys [13], aluminum alloys [14], steels [15], superalloys [16], high-entropy alloys [17], and shape memory alloys [2]. Many scholars have reported the preparation of Fe-Mn-Si shape memory alloys by LPBF [5,18,19,20]. Ferretto et al. [18] reported the first preparation of the Fe-17Mn-5Si-10Cr-4Ni alloy by LPBF. Their superelasticity and shape memory properties exceed those of Fe-Mn-Si shape memory alloys prepared by the as-cast process. At the same time, they demonstrated the shape memory effect of Fe-Mn-Si parts with complex structures. They also found that the microstructure of Fe-Mn-Si shape memory alloys can be modified via LPBF process parameter [5]. By varying the scan speed, samples characterized by coarse elongated grains with strong <001> orientation along the build direction or by finer equiaxed grains without preferential orientation can be fabricated. Kim et al. [19] investigated the phase transformation behavior from metastable BCC to FCC in Fe-Mn-Si-based shape memory alloy processed by LPBF. They found that the phase compositions in Fe-Mn-Si shape memory alloys are very different at different laser energy densities. Fabricating Fe-Mn-Si SMAs via LPBF produces parts with more complex structures, which is an advantage that allows for the development of more potential application scenarios.

However, the main applications for Fe-Mn-Si SMAs are currently in the construction sector, including the manufacture of pre-stressed concrete [21] and seismic isolation systems [22], where the parts used tend to be large in size and relatively simple in shape. Therefore, the LPBF process is not very suitable for these applications. A conspicuous gap in the literature becomes evident upon review: the application of laser-directed energy deposition (LDED) in the creation of Fe-Mn-Si SMAs remains uncharted territory. The LDED process is capable of preparing large components and is particularly suitable for application scenarios of Fe-Mn-Si SMAs [21,22,23,24]. This absence of research is particularly striking given the known advantages of LDED, including superior control over thermal gradients, potential for high cooling rates, and, most importantly, ease of preparation of large parts.

This study seeks to pioneer research in this unexplored domain, initiating a comprehensive examination into the shape memory effects of LDED-processed Fe-Mn-Si-based alloys. We will delve into the microstructural evolution corresponding to different levels of deformation and draw correlations with their shape memory behavior. By venturing into this novel area, our research is set not only to augment the academic corpus regarding Fe-Mn-Si-based SMAs but also to catalyze advancements in their practical applications, leveraging the untapped potential of LDED in the realm of SMAs.

## 2. Methods

The alloy was prepared with a mass ratio of Fe-20.2Mn-5.6Si-8.9Cr-5.0Ni using materials of pure Fe (99.9%) ingots, pure Mn (99.9%) ingots, pure Si (99.9%), pure Cr (99.9%) ingots, and pure Ni (99.9%) ingots. The Fe-Mn-Si alloy powder was prepared using the vacuum induction melting gas atomization method. The specific chemical composition of the powder was determined using the inductively coupled plasma optical emission spectrometry method (SPECTROBLUE SOP, SPECTRO, Kleve, Germany), as shown in Table 1. In this study, two types of LDED techniques were selected to form the Fe-Mn-Si-based alloy. The LDED forming used the coaxial powder-feeding laser melting equipment (RC-LDM8060, Zhongkeyuchen, Nanjing, China). To prevent the powder and samples from being oxidized during the forming process, argon was introduced into the forming cabin for protection, controlling the oxygen content below 100 ppm and the water content below 500 ppm. The substrate used for the alloy sample forming was 304 steel, which has a thermal expansion coefficient similar to that of the Fe-Mn-Si-based alloy, conducive to the wetting of the metal droplets on the substrate. A 50 × 50 × 30 mm^3^ cubic sample was deposited on the substrate. The optimized LDED parameters of 1000 W laser power, 800 mm/min scanning speed, 0.3 mm layer thickness, and 1 μm hatch spacing were selected in this study [25].

The pre-alloyed powder and as-deposited samples were analyzed for phase composition using an X-ray diffractometer (XRD, D/max 2500VB type, Isuzu, Yokohama, Japan) with Cu-Kα radiation. The selected diffraction angle range was 20~100°, and the scanning speed was 5°/min.

Metallographic samples were prepared using ASTM standard methods. After the samples were prepared, they were etched with a metallographic etching solution (1 g FeCl_3_ + 20 mL HCl + 60 mL H_2_O) for 15~20 s to show the microstructural features of the as-deposited samples. The plane cracks, porosity, melt pool, scan track features, and grain morphology were observable under an optical microscope (OM, Leica DM2700P, Wetzlar, Germany). The microstructure characteristics of the alloy samples were observed using a scanning electron microscope (SEM, Quanta FEG 250, Fremont, CA, USA) equipped with an energy dispersive spectrometer (Energy Disperse Spectroscopy, EDS). The main element distribution of the alloy was quantitatively analyzed using an emission electron probe microanalyzer (EPMA, JXA-8539F, JEOL Ltd., Tokyo, Japan). Electron backscatter diffraction (EBSD, Helios Nanolab G3 UC, FEI, Hillsboro, OR, USA) was performed on the samples, mainly analyzing the grain orientation and size characteristics, martensite phase, and austenite phase distribution features.

Room temperature tensile tests on the alloy samples were conducted using an MTS universal mechanical tensile testing machine at a strain rate of 10^−3^/s. The dog-bone-shaped tensile specimens with a total length of 21 mm and a thickness of 1.5 mm were created from LPBF printing blocks. The gauge length and width were 8 and 2.5 mm, respectively. The tensile properties of the specimens with the loading direction along the scanning direction (X) and perpendicular to the scanning direction (Y) and the building direction (Z) were tested 3 times each.

**Figure 1:**
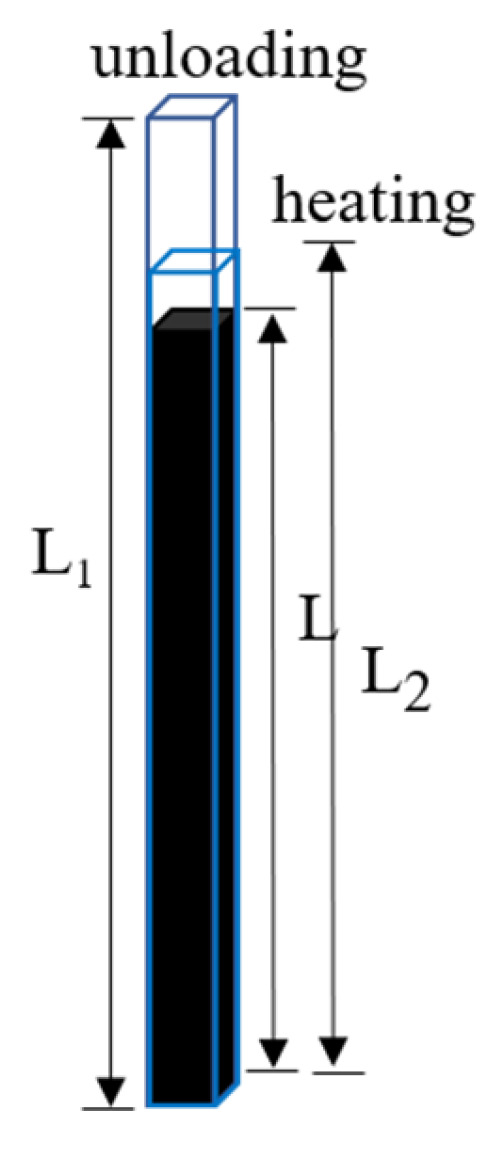
Shape recovery rate test schematic diagram of tensile method.

The shape memory properties of the alloy were tested using tension methods as shown in Figure 1. The tensile specimen of length *L* underwent a certain amount of tensile pre-deformation ε_0_ after loading, and the length *L*_1_ after unloading was measured. It was then heated to 400 °C and held for 15 min, and after cooling to room temperature, the length *L*_2_ of the specimen was measured. The shape recovery rate *η* can be obtained according to Formula (1):

(1) $$\eta =\frac{L_{1}-L_{2}}{L_{1}-L}\times 100\%$$

Recoverable deformation is defined as ε_0_ multiplied by *η*.

## 3. Results and Discussion

### 3.1. Microstructure and Phase Constituent

**Figure 2:**
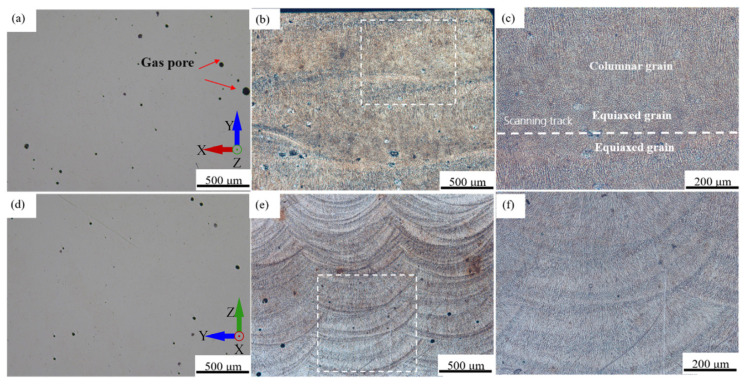
Optical micrographs of plane morphology and internal microstructure of as-deposited alloy: (**a**) horizontal plane of LDED sample; (**b**) scanning track; (**c**) grain morphology in the scanning track; (**d**) vertical plane of LDED sample; (**e**) molten pool; (**f**) grain morphology in molten pool.

The plane morphology of the horizontal and vertical faces of the LDED-formed Fe-Mn-Si-based alloy, as well as the organizational features after etching, are shown in Figure 2. From Figure 2a,d, it can be seen that there are pores of different sizes inside the alloy. This is due to the high temperature inside the melt pool during the forming process, which causes the water vapor and the gas generated by Mn volatilization. This part of the gas does not have time to escape, forming spherical pores in the melt pool. Moreover, no obvious cracks are observed inside the alloy, possibly because the residual stress from the forming process drives the martensitic phase transformation, effectively releasing it and suppressing the formation and expansion of cracks.

From Figure 2b, it can be observed that the width of the scan track is about 1 mm, which is basically consistent with the scan spacing of 1 mm, indicating that the overlap effect between the scan tracks is good. Figure 2c shows that the inside of the scan track has a typical equiaxed-columnar mixed crystal structure. Equiaxed crystals are mainly distributed at the boundaries of the scan tracks, while columnar crystals grow along the scan track boundaries inward and outward. This is because when a new scan track is formed, the alloy melt at the boundary of the scan track can dissipate heat to the adjacent formed track and substrate, cooling more rapidly and thus forming equiaxed crystals. With the formation of boundary equiaxed crystals, the cooling speed of the alloy melt inside the scan track slows down, forming a larger temperature gradient, which gives the solidifying melt a certain directionality, leading to the formation of columnar crystals.

The width of the melt pool is between 1.2 and 1.5 mm, the depth of the melt pool is between 0.1 and 0.5 mm, and the layer thickness is 0.3 mm (Figure 2e). This is due to the repeated melting of the deposition layers, and there will be overlap between layers.

Figure 2f shows that the bottom and top of the melt pool are equiaxed crystals, and the columnar crystals inside the melt pool grow outward from the bottom of the melt pool. During the LDED process, a transformation from columnar to equiaxed crystals occurs at the top of the melt pool, known as the columnar to equiaxed transition (CET) [26]. This is due to the specific heat transfer and solidification mechanisms of the melt pool itself. The bottom of the melt pool cools extremely quick; the supercooling at the forefront of the solid–liquid interface is greater than the nucleation supercooling, and the nucleation rate is high, forming equiaxed crystals. Because of the large thermal gradient (G), grains grow outward along the boundary of the melt pool to form columnar crystals. As the solidification process proceeds, G from the bottom to the top of the melt pool gradually decreases, while the solidification speed gradually increases, and the G/R value decreases accordingly [26]. At the same time, the direction of heat dissipation also changes from originally being perpendicular to the top to being close to parallel to the scanning direction, so equiaxed crystals appear at the top of the melt pool [27].

**Figure 3:**
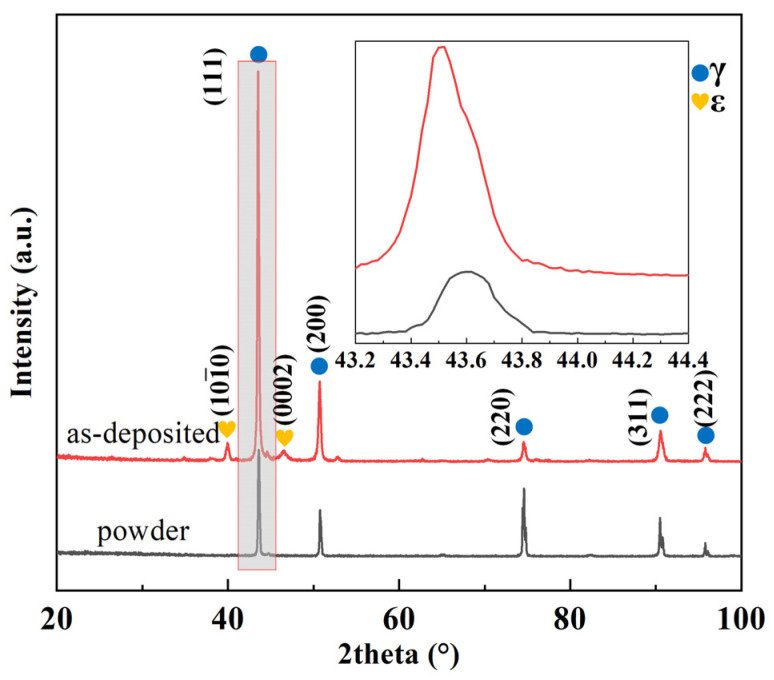
X-ray diffraction patterns of the Fe-Mn-Si pre-alloyed powder and the as-deposited sample.

Figure 3 shows the phase distribution diagrams of the alloy powder and the deposited alloy. It is clear from the diagram that the alloy powder has a single-phase structure of γ-austenite, while the deposited Fe-Mn-Si alloy contains two phases, γ-austenite and ε-martensite. During the LDED process, layer-by-layer thermal cycling can produce large residual stress in the as-deposited materials [23,28]. Under these circumstances, ε-martensite can be formed by stress-induced martensitic transformation with the assistance of the residual stresses in the as-deposited samples [29].

Furthermore, from the inset in Figure 3, it can be observed that the (111)_γ_ diffraction peak in the deposited alloy is shifted toward a smaller angle compared to the (111)_γ_ diffraction peak in the powder. This phenomenon is probably due to the evaporation of Mn during the LDED process, which leads to an increase in the interplanar spacing of γ-austenite. According to Bragg’s diffraction equation, 2dsin θ = nλ (where d is the interplanar spacing, θ is the angle of the diffraction peak, and λ is the wavelength of the X-ray), the increase in interplanar spacing leads to an increase in the diffraction angle, meaning the diffraction peak shifts to a higher angle.

**Figure 4:**
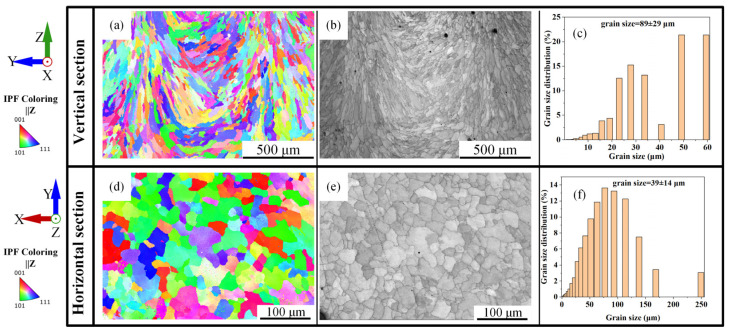
EBSD analysis of vertical plane (**a**–**c**) and horizontal plane (**d**–**f**) of as-deposited sample: (**a**,**d**) inverse pole figure maps; (**b**,**e**) grain image quality maps; (**c**,**f**) grain size distribution charts.

To investigate the morphology, size, and orientation characteristics of the grains in the as-deposited Fe-Mn-Si alloy, EBSD analysis was performed on the horizontal and vertical planes, as depicted in Figure 4. Figure 4a presents an inverse pole figure (IPF) map of the grains in the vertical planes. The insert figure is the inverse pole figure corresponding to the building direction. It is evident from the figure that the columnar grains on the vertical plane exhibit preferred growth orientations of <001> and <101>, with the <001> orientation being more pronounced. Columnar crystal growth typically has a preferred orientation, which is generally closest to the thermal gradient direction. For materials with an FCC structure, the preferred orientation of columnar crystals is usually in the <100> directions, and grains tend to grow preferentially in the <100> direction that forms the smallest angle with the scanning direction [30]. Consequently, the columnar crystals on the build plane during the deposition process exhibit a <001> preferred growth orientation.

Figure 4d shows an IPF map of the grains in the horizontal plane. The insert figure is the inverse pole figure corresponding to the scanning direction, indicating that the equiaxed grains do not have a specific preferred orientation. This is attributed to the lack of a specific preferred orientation in the growth of equiaxed grains. Figure 4b,e clearly demonstrates the morphological characteristics of the equiaxed and columnar crystals, with a more uniform distribution of grain size in the equiaxed crystal region compared to that in the columnar crystal region.

Figure 4c,f reveals that the average grain sizes of the vertical plane and horizontal plane samples are 89 μm and 39 μm, respectively. The grain size on the horizontal plane is comparatively smaller, primarily below 60 μm, and more evenly distributed.

These analyses indicate that there are certain differences in the microstructural organization at different parts of the deposited samples, which in turn would result in variations in properties.

**Figure 5:**
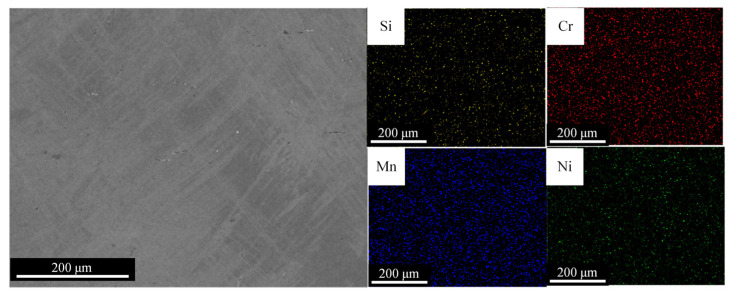
Elemental distribution of the as-deposited alloy in a large area via SEM-EDS.

**Figure 6:**
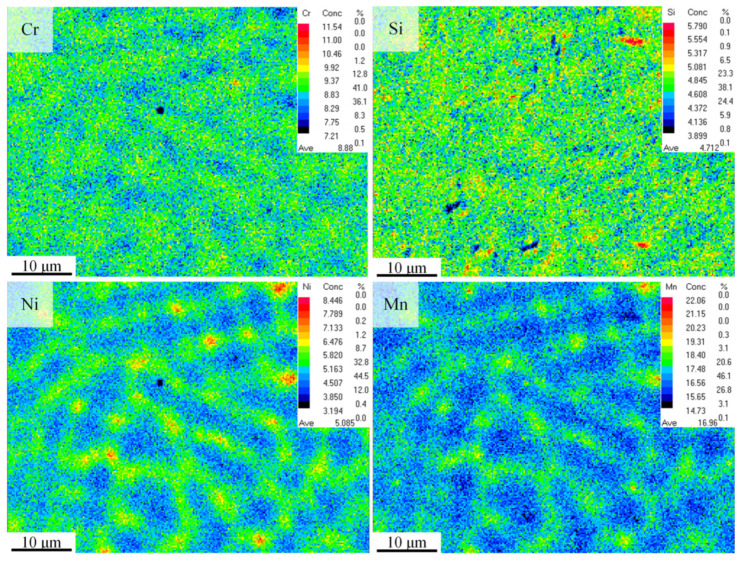
Element distribution of the as-deposited sample within several grain ranges via EPMA.

The shape memory properties of Fe-Mn-Si-based alloys are significantly influenced by changes in alloy composition, with the uniformity of the alloy composition being a key factor in ensuring stable properties. To study the distribution of elements within the Fe-Mn-Si-based alloy formed by LDED, SEM-EDS and EPMA were performed on the alloy, as shown in Figure 5 and Figure 6. Figure 5 shows that the alloy’s elements are evenly distributed over a large range, with no regional segregation. This is due to the LDED-forming process, where the cooling rate reaches 10^3^~10^5^ K/s [23,31], suppressing element segregation to a certain extent. Compared to traditional forming techniques such as casting (with a cooling rate around 10 K/s), samples formed by LDED technology do not require prolonged heat treatment to ensure the uniformity of the alloy’s composition.

Figure 6 displays the distribution of alloy elements within several grain ranges, showing that Cr and Si are also fairly evenly distributed within the grain scale. The average content of the measured Cr is 8.88%, which is essentially consistent with the designed composition. The distribution of Ni and Mn elements shows that their content within the grains is lower than at the grain boundaries, indicating grain boundary segregation. This may be because the diffusivity of Ni and Mn atoms in the crystal is weaker than at the grain boundaries, leading to grain boundary segregation [32]. The content of Mn is around 17%, which differs significantly from the 20.2% in the nominal composition, possibly due to the high saturation vapor pressure and low boiling point of Mn, resulting in its volatilization during the forming process [33]. Microsegregation is possible during the laser additive manufacturing process but can be effectively eliminated with simple heat treatment.

### 3.2. Mechanical and Shape Memory Properties

**Figure 7:**
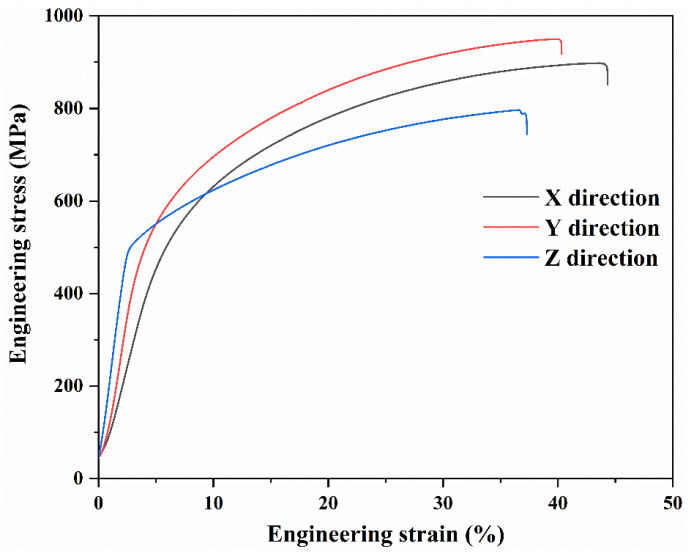
Stress-strain curves of the as-deposited samples in horizontal plane and vertical plane.

To explore the mechanical properties of the deposited Fe-Mn-Si alloy, the tensile properties of the samples in the scanning direction (X) and perpendicular to the scanning direction (Y) and building direction (Z) were tested, and the results are shown in Figure 7. From the figure, it can be seen that the tensile strength of the samples in the horizontal directions is essentially the same. The tensile strength and elongation in the X direction are 892 ± 28 MPa and 43 ± 2%. The samples along the Y direction have a tensile strength and elongation of 933 ± 51 MPa and 42 ± 5%. The samples in the Z direction have a tensile strength and elongation of 797 ± 47 MPa and 38 ± 4%, respectively.

The tensile strength of the samples on the horizontal plane is significantly higher than that of the samples on the vertical plane, while the elongation is not much different. The mechanical properties of the samples on the horizontal plane are better than those of the samples on the vertical plane, possibly because there are more defects such as pores on the vertical plane, which are prone to crack formation during tension, leading to fracture under smaller stress. Moreover, as known from the previous section, the average grain size of the samples on the horizontal plane is smaller than that of the samples on the vertical plane, which is also a reason for the better mechanical properties of the horizontal plane samples.

Generally speaking, as the strength of an alloy increases, its ductility tends to decrease. However, due to the stress-induced martensitic phase transformation characteristics of the Fe-Mn-Si-based shape memory alloy, the external force it receives can serve as the driving force to induce the ε-martensite phase transformation. When the alloy is subjected to a small external stress, the deformation is mainly due to the strain caused by the stress-induced martensitic transformation. The martensite formed by the phase transformation is a strengthening phase relative to the parent austenite phase, thereby enhancing the strength.

As the stress increases, the undeformed austenite continues to transform into martensite under the drive of stress, and strain is produced accompanying the martensitic phase transformation. The more thorough the martensitic phase transformation, the greater the deformation caused by the stress-induced martensitic transformation. Simultaneously, as the external stress increases, the dislocation slip will produce irreversible plastic deformation, ensuring good ductility of the alloy. The more martensite present, the better its effect on strengthening the matrix, and with the tangling and accumulation of dislocations, the driving force required for subsequent martensitic transformation and dislocation slip increases, and the strength of the alloy also increases. When the stress increases to a certain extent, the closely packed hexagonal ε-martensite undergoes twinning deformation, producing deformation twins while also accompanied by certain deformation, but the direct contribution of the twin deformation to the alloy deformation is relatively small [4,34]. Twinning deformation changes the orientation of the crystal, making some slip systems that were originally unfavorable in the crystal shift to directions favorable for slipping, thereby stimulating further slip and crystal deformation. Slipping and twinning alternately proceed, promoting each other, allowing the crystal to produce greater plastic deformation.

**Figure 8:**
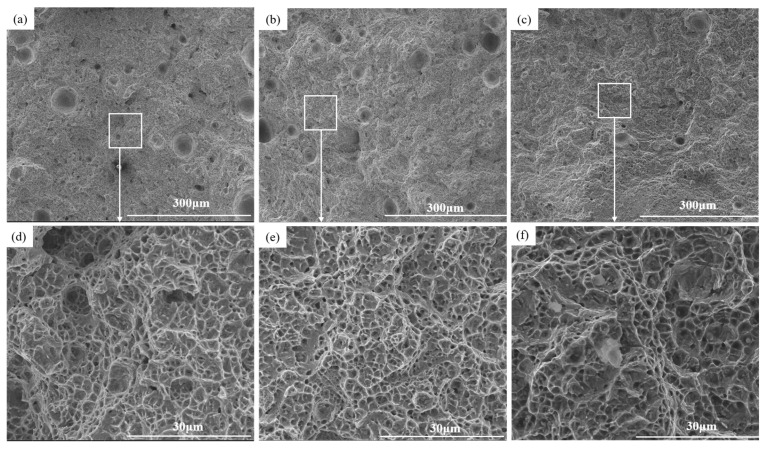
SEM micrographs showing fracture morphologies of the as-deposited samples in different directions: (**a**) X direction; (**b**) Y direction; (**c**) Z direction; (**d**) the area selected in (**a**); (**e**) the area selected in (**b**); (**f**) the area selected areas in (**c**).

Figure 8 shows the fracture morphology of tensile specimens in different directions. From the figure, it can be seen that there are numerous dimples at the fracture sites of all three specimens. A dimple fracture is an indication of a ductile fracture, suggesting that the alloy possesses good ductility. When the alloy is subjected to tensile deformation, tiny pores within the alloy and the interfaces between martensite and austenite initially crack to form the source of dimples. As the stress increases and the strain grows, the dimples gradually tear open, forming raised tearing ridges around them, indicating a high degree of plastic deformation. The tearing ridges in the figure appear bright in contrast.

**Figure 9:**
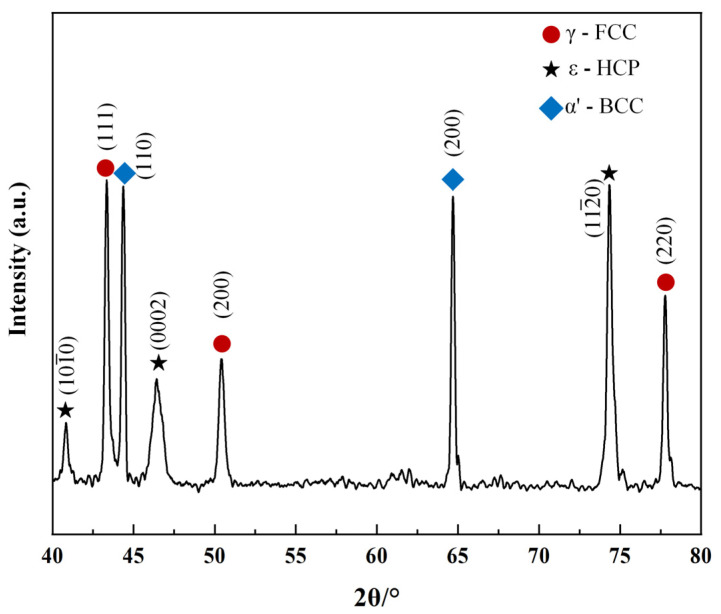
X-ray diffraction pattern of samples after 43% tensile deformation.

The description in Figure 9 illustrates the phase transformations of the Fe-Mn-Si alloy after tensile deformation. The graph reveals that the phases of the deformed alloy mainly include γ-austenite (FCC), ε-martensite (HCP), and α′-martensite (BCC). Among them, γ-austenite, serving as the matrix phase, transforms into ε-martensite under external stress. With increasing stress, both the content and size of ε-martensite increase, and ε-martensite formed at different times may have different orientations, leading to collisions within the crystal [4]. α′-martensite forms at the intersections of these collisions. This transformation mainly follows the sequences from γ-austenite to ε-martensite and from γ-austenite to ε-martensite to α′-martensite. Notably, α′-martensite results from the transformation of ε-martensite under external force, not a direct transition from γ-austenite [35,36].

Moreover, by comparing the height of the strongest peak for each phase in the X-ray diffraction, we can qualitatively determine that after substantial deformation, the alloy primarily contains the martensitic phase, with a higher content of α′-martensite than ε-martensite. This suggests a significant collision phenomenon among ε-martensite.

The substantial presence of martensite in the alloy indicates that under stress, a large part of the stress is used to drive the martensitic transformation. This martensitic transformation contributes to a considerable plastic deformation in the alloy. Simultaneously, as a strengthening phase, martensite not only enhances the strength of the matrix but also further improves the strength by inhibiting the movement of dislocations.

Therefore, the deposited Fe-Mn-Si-based alloy demonstrates excellent mechanical properties, encompassing both high strength and good plasticity. This offers immense potential for its use in various engineering applications, especially those requiring high strength and plasticity.

**Figure 10:**
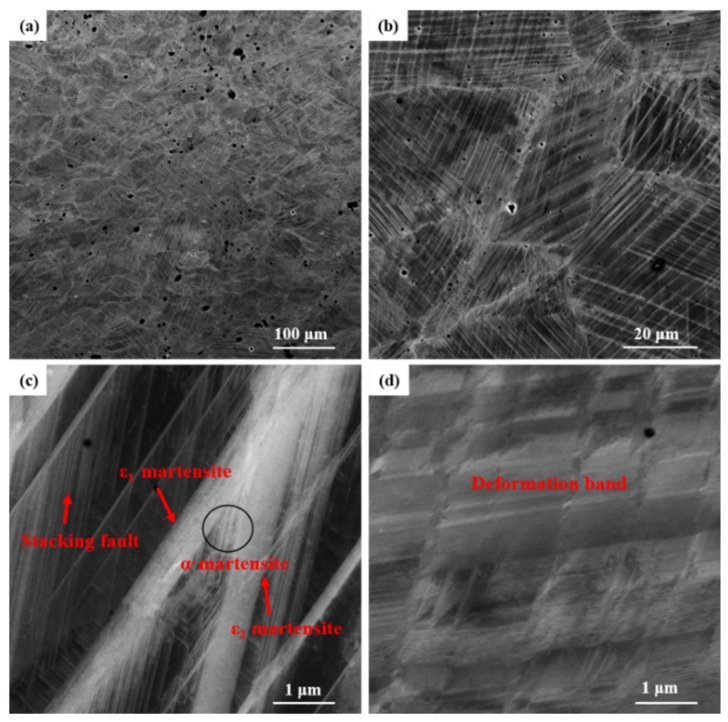
SEM morphology of the sample after 43% tensile deformation: (**a**) grain morphologies; (**b**) martensite distribution in grains; (**c**) morphology of ε-martensite and stacking faults; (**d**) deformation bands.

Figure 10 shows the microstructure inside the alloy after being subjected to a 43% deformation through tensile testing. From Figure 10a, it can be observed that a large number of lath-shaped martensites are distributed within the grains of the deformed samples. Figure 10b is an enlarged view of the region in Figure 10a, revealing that under large deformation, the grains contain ε-martensite with different orientations.

Figure 10c is an enlarged view of a portion of the region in Figure 10b, from which the various thicknesses of the lath-shaped martensite can be clearly seen. Stacking faults, under the action of external forces, form ε-martensite with the expansion of Shockley partial dislocations and are also considered to be fine ε-martensite with only two atomic layers [34]. As the stress increases, martensites of different orientations continue to grow under external stress and interact with each other, as evidenced by the ε_1_-martensite and ε_2_-martensite crossing and colliding at the junction to form α′-martensite, accompanied by a certain amount of irreversible plastic deformation [37].

When the temperature rises above the austenite transformation temperature, the ε-martensite immediately reverse transforms into γ-austenite, while the α′-martensite requires a higher temperature to transform back into γ-austenite, and the deformation induced by it is irreversible [38]. Figure 10d depicts the deformation bands formed in the grains under larger stress, which generally contain a higher dislocation density [39].

**Figure 11:**
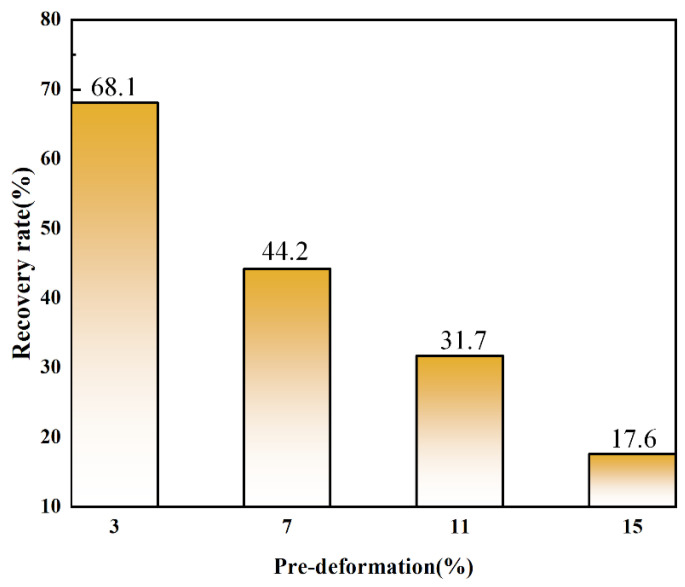
Shape recovery rates of the as-deposited samples with different pre-deformations.

To study the shape memory properties of deposited Fe-Mn-Si-based alloys, the tensile method was used to measure the shape memory effect of the alloy under different deformation amounts, with the results shown in Figure 11. The tensile test results indicate that the shape recovery rates corresponding to tensile deformations of 3%, 7%, 11%, and 15% were 68.1%, 44.2%, 31.7%, and 17.6%, respectively, with the recoverable deformation amounts being 2.04%, 3.09%, 3.49%, and 2.64%. The shape recovery rate also decreased with increasing deformation.

In summary, the maximum recoverable deformation of the LDED-formed Fe-Mn-Si-based shape memory alloy measured in this study is 3.49%, which is higher than the shape memory performance of traditionally deformed polycrystalline Fe-Mn-Si shape memory alloys (<3%) [6]. The improvement in shape memory performance is related to the large number of stacking faults distributed in the LDED Fe-Mn-Si-based shape memory alloy because the stacking faults can transform into ε-martensite under stress induction [34,37]. The larger the stacking fault density, the smaller the driving force required for stress-induced martensite. The more ε-martensite is produced under the same stress conditions, the more it benefits the improvement of shape memory performance [40].

Additionally, the as-deposited Fe-Mn-Si alloy contains pre-existing ε-martensite, which can serve as the nucleation site for stress-induced martensite. When loaded, it can expand and reduce the stress required to induce new martensite [41]. Pre-existing ε-martensite can also inhibit dislocation movement and reduce plastic slippage. Moreover, due to the faster cooling rate during the LDED forming process, the grains of the deposited alloy are smaller [23]. According to the Hall–Petch formula, the smaller the grain size, the greater the critical stress required for the dislocation slip. Therefore, inside the grains of the as-deposited alloy, the dislocation slip is more challenging, and the amount of irreversible deformation caused by the dislocation slip is relatively small, with deformation mainly borne by stress-induced martensitic transformation. Considering these factors, the LDED-formed Fe-Mn-Si alloy has good shape memory performance.

## 4. Conclusions

In conclusion, this study provides an in-depth exploration of the microstructure, mechanical properties, and shape memory properties of an Fe-Mn-Si-based alloy fabricated via LDED. Through a comprehensive series of analyses, including X-ray diffraction, microscopic observation, and tensile testing, several critical findings and advancements have been highlighted:

1. The internal phase transformations of the alloy were meticulously analyzed. Upon tensile deformation, it was observed that the alloy underwent substantial structural changes; chiefly among these was the transformation from γ-austenite to ε-martensite and eventually to α′-martensite. These transformations play a pivotal role in the shape memory effects and overall mechanical performance.
2. The microstructure of the alloy post-deformation was investigated, revealing a high volume of various martensitic structures and stacking faults, particularly within grains that have undergone significant deformation. The presence of these structures, especially the ε-martensite, which forms from stacking faults, was deemed crucial for the shape memory properties of the alloy.
3. Tensile testing yielded critical data on the shape memory effects of the alloy under various degrees of deformation. As deformation increased, the shape recovery rate diminished. However, notably, the LDED-formed Fe-Mn-Si-based shape memory alloy exhibited a maximum recoverable strain of 3.49% under a pre-deformation of 11%, a value superior to that of traditionally processed Fe-Mn-Si shape memory alloys.
4. The rapid cooling rate during the LDED process resulted in finer grains in the deposited alloy, subsequently increasing the critical stress required for dislocation slip according to the Hall–Petch relationship. This phenomenon makes the dislocation slip more challenging within the grains of the deposited alloy, thereby reducing irrecoverable deformation and enhancing the shape memory effect.

## Figures and Tables

**Table 1:** The elemental composition of the Fe-Mn-Si alloy powder.

Element	Fe	Mn	Si	Ni	Cr
Concentration (wt%)	60.8	20.0	5.5	4.9	8.8

## Data Availability

The data presented in this study are available on request from the corresponding author.
